# Fabrication and Testing of Multi-Hierarchical Porous Scaffolds Designed for Bone Regeneration via Additive Manufacturing Processes

**DOI:** 10.3390/polym14194041

**Published:** 2022-09-27

**Authors:** Carmen M. González-Henríquez, Fernando E. Rodríguez-Umanzor, Nicolas F. Acuña-Ruiz, Gloria E. Vera-Rojas, Claudio Terraza-Inostroza, Nicolas A. Cohn-Inostroza, Andrés Utrera, Mauricio A. Sarabia-Vallejos, Juan Rodríguez-Hernández

**Affiliations:** 1 Departamento de Química, Facultad de Ciencias Naturales, Matemáticas y del Medio Ambiente, Universidad Tecnológica Metropolitana, Santiago 7800003, Chile; 2 Programa Institucional de Fomento a la Investigación, Desarrollo e Innovación, Universidad Tecnológica Metropolitana, Santiago 8940000, Chile; 3 Research Laboratory for Organic Polymer (RLOP), Facultad de Química y Farmacia, Pontificia Universidad Católica de Chile, Santiago 7810000, Chile; 4 Laboratorio de Nanobiomateriales, Instituto de Investigación en Ciencias Odontológicas, Facultad de odontología, Universidad de Chile, Santiago 8380544, Chile; 5 Departamento de Ingeniería Mecánica, Facultad de Ingeniería, Universidad de Santiago de Chile, Santiago 9170124, Chile; 6 Facultad de Ingeniería, Arquitectura y Diseño, Universidad San Sebastián, Santiago 8420524, Chile; 7 Polymer Functionalization Group, Departamento de Química Macromolecular Aplicada, Instituto de Ciencia y Tecnología de Polímeros-Consejo Superior de Investigaciones Científicas (ICTP-CSIC), 28006 Madrid, Spain

**Keywords:** bone scaffold, additive manufacturing, salt leaching, wrinkled micropatterns, ATRP synthesis

## Abstract

Bone implants or replacements are very scarce due to the low donor availability and the high rate of body rejection. For this reason, tissue engineering strategies have been developed as alternative solutions to this problem. This research sought to create a cellular scaffold with an intricate and complex network of interconnected pores and microchannels using salt leaching and additive manufacturing (3D printing) methods that mimic the hierarchical internal structure of the bone. A biocompatible hydrogel film (based on poly-ethylene glycol) was used to cover the surface of different polymeric scaffolds. This thin film was then exposed to various stimuli to spontaneously form wrinkled micropatterns, with the aim of increasing the contact area and the material’s biocompatibility. The main innovation of this study was to include these wrinkled micropatterns on the surface of the scaffold by taking advantage of thin polymer film surface instabilities. On the other hand, salt and nano-hydroxyapatite (nHA) particles were included in the polymeric matrix to create a modified filament for 3D printing. The printed part was leached to eliminate porogen particles, leaving homogenously distributed pores on the structure. The pores have a mean size of 26.4 ± 9.9 μm, resulting in a global scaffold porosity of ~42% (including pores and microchannels). The presence of nHA particles, which display a homogeneous distribution according to the FE-SEM and EDX results, have a slight influence on the mechanical resistance of the material, but incredibly, despite being a bioactive compound for bone cells, did not show a significant increase in cell viability on the scaffold surface. However, the synergistic effect between the presence of the hydrogel and the pores on the material does produce an increase in cell viability compared to the control sample and the bare PCL material.

## 1. Introduction

Bone and cartilage injuries beyond the self-repair threshold represent a significant challenge [1]. Impact and torsional joint loading can injure the bone tissue and its surroundings, causing various medical complications, such as joint dysfunction or progressive degeneration [2]. Bone defects, comminuted fractures, and other orthopedic diseases have complex, painful, and extended treatments. With a worldwide aging population and the growing problem of obesity, acute cases of bone and cartilage injuries are estimated to increase rapidly in the upcoming years [3]. Due to the large area of bone tissue and the limited range of their self-recovery mechanism, external implants are needed to replace the original bone in some critical cases [3]. Artificial bone-engineered alternatives were proposed to overcome these problems via tissue engineering (TE) alternatives [4]. TE was defined in 1993 by Langer and Vacanti as “an interdisciplinary field that aims at the development of biological substitutes that can be used to replace, restore or improve tissue function” [4]. TE strategies typically involve three main components: scaffolds, cells (differentiated or undifferentiated), and biological signaling molecules [5]. Consequently, TE strategies, such as scaffold design, fabrication, and optimization for bone regeneration with specific chemical or physical functionalities, have emerged as an effective solution to the problem of bone replacement.

A bone scaffold should promote cell migration, which is essential for proper vascularization and bone in-growth [6]. As a result, the material must be self-degradable without producing toxic byproducts to be adaptable as a bone scaffold. For bone scaffold fabrication, composites of hydroxyapatite (HA) and polycaprolactone (PCL) have been widely used in recent years [7]. HA is similar to native bone (70% structural similarity) and has been incorporated into various biomedical devices due to its osteoconductive and biocompatible properties [8]. However, their brittle nature limits their use as a scaffold for bone applications. PCL is a biocompatible and biodegradable polymer commonly used for biomedical applications such as bone scaffolds or long-term implantable drug delivery systems [9]. Thus, PCL is generally used together with HA to provide the appropriate mechanical strength required for scaffold performance [10]. For the fabrication of bone scaffolds using PCL/HA composites, various fabrication methods, such as salt leaching [11], thermally induced phase-separation (TIPS) [10], and 3D printing [3,9,12], have been employed in the last years.

Briefly, salt leaching is a fabricating method to create a porous structure by pouring composites mixed with porogen particles into a mold to make a specific shape and then rinsing it in water to remove the porogen particles; thus, leaving pores in the structure [9]. Additive manufacturing (AM), commonly known as 3D printing, is defined by the ISO/ASTM standards as the “process of joining materials to make parts from 3D model data, usually layer upon layer” [13]. This type of technology has experienced enormous growth in the last decade, particularly for biomedical device fabrication, being applied in multiple areas, ranging from human-made objects that provide physical support, such as knee implants and synthetic blood vessels, to applications that improve the functionality of human organs. Typical manufacturing processes, such as organic foam impregnation, freeze-drying, gas foaming, and sol-gel methods, present difficulties when manufacturing scaffolds with a customized structure and complex internal/external shapes [14]. Accordingly, one of the main advantages of AM technologies, when it comes to scaffold fabrication, is the possibility of not only designing the external shape of the printed part to fit perfectly in the patient but also precisely defining the internal structure of the material according to the requirement necessities for the intended application [15]. For example, Sohrabian et al. analyzed the role that the inner scaffold architecture in the mechanical properties of the printed part plays [16], concluding that only by varying the internal structure of the 3D printed part is it possible to increase its Young modulus by more than 47%.

Another crucial issue to consider when designing the scaffold is the overall material biocompatibility. To improve material biocompatibility, two paths can be followed. On the one hand, chemical alterations can be carried out on the material’s surface, and on the other hand, morphological modifications or structurations could be induced on the surface [17]. Several conventional methodologies could create surface microstructures, such as lithography, hot-embossing, or electron beam technologies [18,19]. These methodologies are commonly expensive and challenging to apply, resulting in high-effort and complex procedures.

In contrast to conventional approaches, microstructuration based on surface instabilities is economical, straightforward, and allows the fabrication of intricate patterns that are somewhat difficult to obtain by traditional methods. Several scientific studies corroborate that the presence of surface microstructures enhances the biocompatible properties of the material [20,21,22], reducing the chances of scaffold body rejection. Micropatterning could be easily accomplished by taking advantage of surface instabilities produced via the strain mismatch generated in multilayered (or gradient) soft film [23,24].

This article aims to combine salt leaching, bioactive particle inclusion, and AM methods to fabricate a cellular scaffold with an intricate and complex network of pores and microchannels to emulate the bone microstructure. Parallel, homogenous wrinkled patterns were spontaneously created on the top of the material [20,25]. A thin layer of a functional hydrogel was used to this end, which upon UV, vacuum, and argon plasma exposure, was modified to generate wrinkled patterns. In this work, polymeric materials, such as polylactic acid (PLA), PCL, high-impact polystyrene (HIPS), and thermoplastic polyurethane (TPU), were modified to be used as a matrix for fabricating filaments suitable for 3D printing. Porogen particles (NaCl) and bioactive compounds (nano-hydroxyapatite, nHA) were added to the filaments to improve both the biological and mechanical performance of the 3D printed part. The main advance of this article is to mix novel scaffold manufacturing technologies (such as 3D printing, salt leaching, and bioactive particle inclusion) with physical and chemical modifications of the material surface via the spontaneous microstructuration of wrinkled surface patterns. To the best of our knowledge, there is no precedent where these technologies were mixed previously to create complex and advanced scaffolds.

## 2. Materials, Equipment, and Methods

### 2.1. Materials

Different polymeric filament types were used in this study: PLA Premium from Raise3D™ (Irvine, CA, USA), PCL (Facilan™ PCL100 natural) from 3D4Makers (Haarlem, The Netherlands), HIPS (M750 natural) from smart materials 3D (Jaen, Spain), and TPU (Ultrafuse 85A) from BASF SE (Ludwigshafen, Germany). Initially, all the filaments were used as purchased. In a second stage, chloroform (CHCl_3_) and N,N-dimethylformamide (DMF) were acquired from Sigma Aldrich (St. Louis, MO, USA) and used to solubilize the PCL for the fabrication and extrusion of the modified filament.

The copolymer designated as *net*-poly(AAc-*co*-AMA) was synthesized via atom transfer radical polymerization (ATRP) using the following reactives. *Monomers*: tert-butyl acrylate (t-BuA, 98%); allyl methacrylate (AMA, 98%); and poly(ethylene glycol) diacrylate (PEGDA), with an average molecular weight of 575 g mol^−1^; these three compounds contain monomethyl ether hydroquinone (MEHQ) as an inhibitor. To extract the inhibitor, these monomers must be purified using an alumina oxide (basic) powder column. These reactives were acquired from Sigma Aldrich (St. Louis, MO, USA) or Merck (Darmstadt, Germany). *Ligand and Initiator*: N, N, N′, N″, N″-pentamethyldiethylenetriamine (PMDETA, 99%) and ethyl α-bromoisobutirate (EBriB, 98%) were used as received. *Copper halides*: copper bromide (CuBr 99,999% trace metals basis) was purchased from Sigma Aldrich (St. Louis, MO, USA). *Solvents and salts:* acetone (ACS reagent ≥ 99.5%), trifluoroacetic acid (TFA, Reagent plus^®^ 99%), dichloromethane (CH_2_Cl_2_), and CHCl_3_ (for analysis EMSURE^®^ ACS, ISO, Reag, Ph Eur), hexane (Reagent plus^®^ 99%), and sodium sulfate (Na_2_SO_4_, Emprove^®^Essential) were all obtained from Merck. The *net*-poly(AAc-*co*-AMA) was used as a base together with 2- hydroxy-4′-(2-hydroxyethoxy)-2-methylpropiophenone (Irgacure 2959, 98.0%) from Sigma–Aldrich (St. Louis, MO, USA) as a photo-initiator, and PEGDA_575_ as a crosslinking agent, for the hydrogel synthesis.

For the nano-hydroxyapatite (nHA) synthesis, ammonium (NH_4_) solution 25%, calcium nitrate tetrahydrate (Ca(NO_3_)_2_*4H_2_O), and ammonium dihydrogen phosphate ((NH_4_)H_2_PO_4_) from Merck (Darmstadt, Germany) were used. Furthermore, sodium chloride (NaCl) particles (acquired from Merck) were sifted using a mesh N° 200 (75 μm opening, Gilson ASTM-U certified) for the filament modification.

For the biological studies, MC3T3-E1 (mouse C57BL/6) cells and a Dulbecco’s Modified Eagle’s Medium (DMEM) with 2-[4-(2-hydroxyethyl)-1-piperazinyl]ethanesulphonic acid (HEPES), penicillin, streptomycin (all from Sigma-Aldrich, St. Louis, MO, USA), and fetal bovine serum Gibco^TM^, from ThermoFisher (FBS, Waltham, MA, USA), were used. Cell viability was determined using the AlamarBlue HS^®^ reagent from ThermoFisher (FBS, Waltham, MA, USA).

Finally, for contact angle measurements, glycerol anhydrous (85%, Emsure^®^) for synthesis and diiodomethane (99%, reagentPlus^®^) were acquired from Sigma Aldrich (St. Louis, Missouri, USA).

### 2.2. Equipment

Proton NMR spectra were acquired using a Bruker Avance II HD 400 operating at 400 MHz (Leipzig, Germany). For the analysis, the copolymers were dissolved in deuterated dimethylsulfoxide (DMSO-d_6_) and deuterated chloroform (CDCl_3_) using tetramethylsilane (TMS) as an internal standard; all these were acquired from ARMAR AG (Döttingen, Switzerland). The average molecular weights (Mw) were determined by size exclusion chromatography (SEC) along with a static light scattering using an Optilab DSP interferometer (from WyattTechnology, Santa Barbara, CA, USA) using tetrahydrofuran (THF) as the mobile phase and a calibration curve constructed using polystyrene (PS) standard samples (molecular weights ranging from 1020 to 1,944,000 g mol^−1^). The SEC measurement was performed using a Dionex P590A liquid chromatography pump equipped with a guard column and two PLgel 5 mm Mixed-C (300 mm × 7.5 mm) columns in series with a Viscotek differential refractometer. The eluent was THF at a flow rate of 1.0 mL min^−1^ at 25 °C. ATR-FTIR was used to determine the chemical composition of the compounds in an FT/IR-4600 spectrometer (JASCO International Co., Ltd., Tokyo, Japan). Raman spectroscopies were carried out in a confocal Raman/AFM spectrometer CRM-Alpha 300 RA (WITec GmbH, Ulm, Germany) with an Nd:YAG laser (max. power 50 mW at 532 nm). The dip-coating deposition was carried out with an automated dipper unit from model VT-04 from MicroTestMachines Co. (Minsk, Belarus).

Photo-polymerizations were carried out through radiation exposure using a UV lamp (9 W) with an emission peak centered at λ = 365 nm from Vilber Lourmat Inc. (Marne-la-Vallée, France). Bresser Trino Researcher II (4–100×) trinocular microscope (Rhede, Germany), coupled with a 5 Mp CCD color camera (Bresser, Rhede, Germany) and with a cold light source Optika CL-41 (Ponteranica, Italy), was used as the first approach for visualizing the topography of the hydrogel wrinkled surface patterns. The hydrogel film topography was obtained using a CoreAFM from Nanosurf Inc. (Woburn, MA, USA) in intermittent contact mode. Additionally, the Force spectroscopy was performed using a tip specially designed for this purpose (PPP-FMR from NanoWorld AG., Liestal, Switzerland). Images were treated using the offline freeware Gwyddion 2.42 [26].

A field emission scanning electron microscope (FE-SEM) model GeminiSEM 360 (Carl Zeiss AG, Oberkochen, Germany), with a Gemini 1 optic InLens detector, which ensures an efficient signal detection for both secondary (SE) and backscattered (BSE) electrons, was used to visualize the material surface. Parallel to FE-SEM imaging, EDX spectroscopy was carried out using an Ultim Max 40 detector (OXFORD Instruments, High Wycombe, UK) to perform elemental analysis on the sample surface. These samples were coated with 8 nm of gold to improve the image resolution using an argon sputter coater (model 108 AUTO, Cressington Scientific Instruments Ltd., Watford, UK). The same sputter coater was also used to generate the surface instabilities, which trigger the spontaneous formation of wrinkled patterns on the hydrogel films.

Water contact angle measurements were performed using a Theta optical tensiometer from Attension-Biolin Scientific (Gothenburg, Sweden), adding 4 μL of liquid phase (water, glycerol, or diiodomethane) over the solid sample.

To determine the hierarchical-porous scaffold’s microstructure, we conducted a micro-CT analysis using a SkyScan 1272 (Bruker Co., Kontich, Belgium). The X-ray source voltage and current were set to 50 kV and 200 µA, respectively. Tomographic scans were acquired using an isotropic voxel resolution of 8 µm per voxel. The 3D images were reconstructed using the NRecon software (Bruker), where misalignment compensation, ring artifact reduction, and hardening filters were employed to improve the image quality.

The EX2 extruder setup (Filabot filament extruder, airpath, and spooler) (Filabot Co., Barre, VT, USA) was used to fabricate the filaments with an extruder nozzle style E (ø = 1.75 mm). An E2 FDM printer from Raise3D technologies Inc. (Irvine, CA, USA) with dual-extrusion technology was employed to print the 3D models.

Two uniaxial single column testing systems were used for this study; (1) model 3343 from Instron (Norwood, MA, USA) was utilized to perform the mechanical tests with a 500 N load cell and a testing speed of 0.05 mm/min, and (2) a testing system Z100 from Zwick/Roell (Ulm, Germany), with a 100kN load cell and a testing speed of 0.5 mm/min.

For biological studies, fluorescence was measured (excitation/emission: 560/590 nm) using a microplate reader BioTek Synergy HTX from Agilent Technologies Inc. (Santa Clara, CA, USA). Furthermore, confocal microscopy was performed on a Nikon C2+ microscope (Melville, NY, USA). Finally, a Zeiss–Colibri epifluorescence microscope (Carl Zeiss AG, Oberkochen, Germany) was used to visualize the cells seeded on the material’s surface.

### 2.3. Methods

#### 2.3.1. Design and Printing of Scaffolds via FDM

Firstly, 3D rectangular pieces of 30 mm × 7 mm × 3 mm were printed using the FDM technique with four different filaments (PLA, PCL, HIPS, and TPU). Afterward, the substrates were treated using a UV lamp ozone generator (UVO) to decrease the contact angle. In this process, a mercury lamp (emission peaks at 185 and 254 nm) generates O atoms simultaneously when atmospheric O_2_ is dissociated via 185 nm radiation, and O_3_ through the association of O_2_ and O mediated by radiation at 254 nm. This continuous generation of O_3_ can be used to chemically modify the surface of polymers; thus, inducing temporary oxidation of the material and, therefore, increasing its wettability by reducing its contact angle.

#### 2.3.2. Copolymer Synthesis

**Figure 1:**
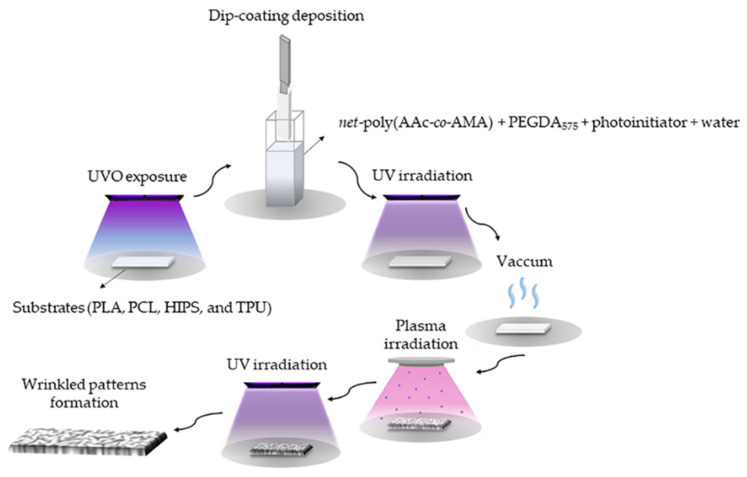
Procedure to generate wrinkled patterns on substrate obtained via FDM technique.

The *net*-poly(AAc-*co*-AMA) was prepared by atom transfer radical polymerization (ATRP) to obtain low polydispersity and controlled chain length. The two-stage procedure is indicated in Figure 1.

Stage 1. Synthesis of *net*-poly(t-BuA-*co*-AMA)

A linear precursor t-BuA/AMA (90:10 ratio) was successfully prepared, similar to those performed by Lejnieks et al. [27]. A three-fold repetition of the experiment proved the reproducibility of the reaction.

The polymerization was performed in a Schlenk flask previously flamed and dried under a vacuum atmosphere (these conditions are general for all polymerizations). The synthesis was carried out using the following stoichiometry:

CuBr (1.11 mmol, 0.16 g), acetone (6 mL), t-BuA (39.0 mmol, 5 g), AMA (4.33 mmol, 0.55 g), PMDETA (1.33 mmol, 0.23 g), and EBriB (1.11 mmol, 0.22 g). This reaction mixture was started by immersing the Schlenk tube in an oil bath at 40 °C, under an argon atmosphere. After 30 h, a dark-green solid is formed and diluted with small amounts of CH_2_Cl_2_ and filtered through a neutral alumina column using CH_2_Cl_2_ as a mobile phase to retain the copper complex (a colorless solution is obtained from this procedure). Posteriorly, the dissolution is concentrated by evaporation, forming a viscous oil. Subsequently, this is dropped into a mixture of H_2_O:CH_3_OH (1:1 ratio), forming a white solid purified by solvent extraction using CHCl_3_ and H_2_O. Finally, Na_2_SO_4_ anhydrous is used to remove traces of water from the organic solution; posteriorly, this dissolution is filtered and concentrated to obtain a white solid. The flask was protected with an aluminum foil to prevent crosslinking of the copolymer. This process generates a copolymer with a tert-butyl group that protects the oxygen, which would be the linkage point for the subsequent formation of the carboxyl group.

Stage 2. Synthesis of *net*-poly(AAc-*co*-AMA)

The remotion of the ter-butyl group was performed by hydrolysis. The copolymer was dissolved in CH_2_Cl_2_ (20 mL), and a certain amount of TFA (4.05 g, 35.5 mmol) was added slowly to the reaction mixture, which was stirred at room temperature for 24 h. Once this time has elapsed, the compound will precipitate in the solid state. If it does not precipitate, the solution must be evaporated to separate the mixture’s components, and thus recover the copolymer. The protected polymer that did not react in the synthesis must be eliminated to purify the copolymer obtained. This procedure was carried out by dissolving the copolymer in THF and then precipitating it in cold hexane. Finally, the solid is dried in a vacuum oven for 24 h at room temperature.

#### 2.3.3. Wrinkles on Different Scaffolds

##### Hydrogel Synthesis and Deposition over the Scaffolds

The reaction mixture was prepared in a glass vial, sealed with a septum, and covered from light with aluminum foil to keep the photo-initiator inactive. A total of 0.0731 g of the *net*-poly(AAc-*co*-AMA) (90:10 ratio) was dissolved in 400 µL of ethanol, 4 g of PEGDA_575_, 1 mL of ultrapure water, and 75 µL of Irgacure 2959 (200 mg in 1 mL of methanol). These were added to the vial and then purged with argon to prevent oxidation.

In parallel, the scaffolds, previously printed via FDM, were exposed to UVO from 20 to 40 min to increase the hydrophilicity of the material. Then, the polymeric mixture was placed on the modified substrate (UVO-treated) via the dip-coating procedure under different deposition conditions, such as input/output speed and immersion cycles. Additionally, each sample was exposed to variable UV irradiation and vacuum times to generate spontaneously wrinkled patterns on the material surface (Table S1 of Supplementary Material).

#### 2.3.4. PCL Filament Modification and Printing of the Porous Structures

##### Incorporation of Porogen and Bioactive Agents in PCL

To obtain a highly porous and bioactive material, NaCl particles (30% *w*/*w*) were incorporated as a porogen agent (particle size ≤ 75 µm) and nHA (particle size ~200 nm) as a bioactive agent at 10% *w*/*w*. nHA was synthesized according to the methodology reported by Karampour et al. [28]. Two parameters (presence/absence of nHA and NaCl) will be optimized to maximize the cell viability and proliferation on the scaffold using the cell line MC3T3-E1 (osteoblasts from mouse C57BL/6). Accordingly, four different filament combinations will be tested to determine the effect of porosity, bioactive agent presence, and hydrogel coverture on cell viability/proliferation in the 3D scaffolds. The detail of each filament composition is shown in Table 1.

To obtain a filament with these agents homogeneously distributed, PCL pellets were dissolved in a solution of CHCl_3_ and DMF (3:1 *v*/*v* ratio). Once the PCL was dissolved, the nHA particles were dispersed in DMF by sonication for 10 min and then added to the PCL solution at 10% *w*/*w*. In parallel, the NaCl particles were added at a concentration of 30% *w*/*w*. The solution was then homogenized for 5 h under constant stirring at 600 rpm and left to stand for 12 h. This process was carried out to obtain a thin film and to favor solvent evaporation. After this time, the material was shredded and dried under vacuum (10^−3^ torr) for 12 h, with the aim of ensuring total solvent extraction. Finally, the PCL-modified filament was prepared using a Filabot extruder with a nozzle diameter of 1.75 mm. The processing parameters for filament preparation were: extrusion temperature = 85 °C, extrusion speed = 35 rpm, maximum cooling ventilation speed, ambient temperature (~19–21 °C), and spooling velocity = 0.75 rpm.

##### Optimization of Printing Parameters and Salt Leaching Process

First, the printing parameters, such as extruder temperature, speed, layer thickness, raster angle, and fill density, were varied depending on each modified filament. From now on, the printed parts will have a cylindrical shape instead of a rectangular one because the radial stress distribution during mechanical tests should be homogenous to avoid possible stress concentrations and reduce fracture risk.

**Figure 2:**
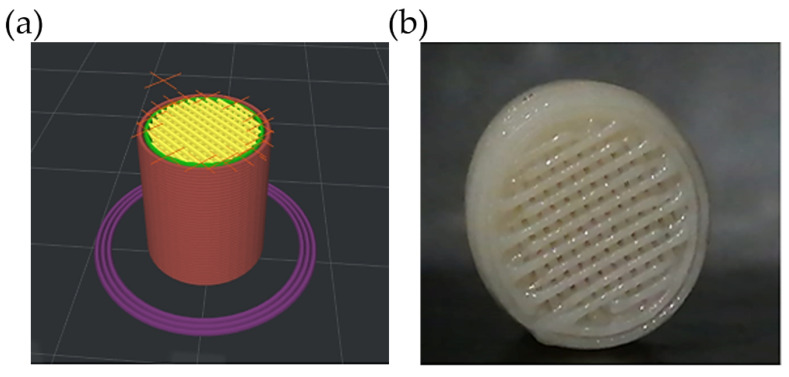
Microchannel visualization in (**a**) computerized 3D design and (**b**) PCL printed scaffold obtained via FDM.

On the other hand, the internal structure of the scaffolds was modified to improve the pores’ interconnectivity and benefit the water permeability during the salt leaching process and cell infiltration. It is worth mentioning that this type of channel favors tissue regeneration and plays an essential role in physicochemical and mechanical signaling to supply the necessary nutrients for cell growth [29,30]. Accordingly, microchannels were incorporated into the computer-aided detection (CAD) model using a filling percentage of 80%, a filling overlay of 45%, and an extrusion speed of 70 mm/s (Figure 2).

Once the scaffolds were printed, the incorporated salt embedded in the composite was leached under continuous stirring in deionized water at room temperature for 14 days. The scaffolds were ozonized (UVO-treated) to improve the hydrogel adhesion on their surface. According to this, the contact angle was monitored at different irradiation times with UVO (0, 5, 10, 15, 20, and 30 min).

#### 2.3.5. Mechanical Tests

Mechanical tests are widely used to obtain the stress–stretch relationship of many materials with the aim of measuring their mechanical properties, such as stiffness (Young modulus) or maximum load fracture. Cylindrical specimens were appropriately measured before the tests, whose initial dimensions were characterized by length (*L*_0_) and radius (*R*_0_). During the test, the load (*F*) and displacement of the jaws (Δ) were recorded. The following equation can determine the stretch (*λ*):

(1) $$\lambda =\frac{L_{0}+\Delta }{L_{0}}$$

Finally, assuming material incompressibility, engineering stress can be calculated as:

(2) $$\sigma =\frac{F}{\pi R_{0}^{2}}\;$$

Pre-loads (5 N) and pre-condition protocols (20 cycles) were used for the mechanical analysis with the aim of avoiding possible pre-stress and geometrical differences related to the manufacturing technique used (FDM printing).

#### 2.3.6. Biological Evaluation (Proliferation Studies)

MC3T3 cells were cultured in DMEM, containing 10% FBS, 10mM of HEPES, 100 U/mL penicillin, and 100 mg/mL streptomycin. The 5 × 10^4^ cells were seeded directly into polymeric printed disks of 10 mm × 3 mm placed in a single well of a 24-well cell culture plate. The medium was replaced every three days, and the cell viability was determined at 1, 3, and 7 days of incubation by using the AlamarBlue HS^®^, a Resazurin-based test, as an indicator of cellular health. After 2 h of incubation with the AlamarBlue HS reagent at 37 °C in a humidified air atmosphere containing 5% CO_2_, the medium was collected from the samples, and fluorescence was measured at an excitation/emission wavelength of 560/590 nm using a microplate reader. MC3T3 cells were seeded onto samples with coverslips (12 mm in diameter) at 50%–70% confluence. Cells were rinsed in phosphate-buffered saline (PBS; 10 mM Tris (pH 7.5), 100 mM NaCl, 5 mM KCl) and then fixed with 4% paraformaldehyde for 10 min at 4 °C. These samples were incubated with DAPI for 10 min and washed five times with PBS, and the samples in coverslips with the cells were mounted in DAKO medium for 5 min. The cells were visualized using a Zeiss–Colibri epifluorescence microscope with a 40× objective. Quantification of the fluorescence was performed using ImageJ software.

## 3. Results and Discussion

The workflow to fabricate these biocompatible scaffolds could be divided into three steps. Firstly, the copolymers, which will be used to create thin microstructured layers on the top of the scaffolds, must be synthesized. Secondly, different 3D printing polymeric materials will be tested as a base to create the scaffolds, and thirdly, the selected material’s composition will be varied with the aim of increasing both the mechanical and the biocompatible properties of the scaffolds.

### 3.1. Characterization of the Copolymers

This work aims to manufacture 3D cellular scaffolds for bone regeneration. Accordingly, our research group fabricated porous pieces with a hierarchical structure using different base materials (substrates) and a series of interconnected micropores and macrochannels formed through CAD modeling and salt leaching processes. Over this substrate, a thin layer of a biocompatible functional copolymer was added, which was then subjected to different external stimuli to spontaneously form wrinkled patterns on its surface that are expected to improve the biocompatibility of the material. Therefore, our first objective was to synthesize and characterize the copolymer used to form wrinkled patterns.

The polymer *net*-poly(AAc-*co*-AMA) was synthesized through the ATRP technique after hydrolysis of the compound *net*-poly(t-BuA-*co*-AMA). Both compounds were characterized by ^1^H-NMR, FT-IR, and Raman spectroscopies to corroborate the synthesis’s success and analyze the materials’ chemical properties. Additionally, the molecular weights and PDI were determined by SEC with traditional calibration.

Figure S1 (Supplementary Materials) shows the ^1^H-NMR spectra of the protected (*net*-poly(t-BuA-*co*-AMA)) and unprotected (*net*-poly(AAc-*co*-AMA)) copolymers in 90:10 ratios, respectively. In the case of the protected copolymer, the analysis shows all the expected signals: δ_a_ (–H_3_) and δ_b_ (–CH_2_–) related to the polymeric backbone. For the allylic groups at δ_d_ = 4.55 ppm (–CH_2_–CH=CH_2_–), δ_e_ = 5.25 ppm (–CH_2_–CH=CH_2_), and δ_f_ = 5.95 ppm (–CH_2_–CH=CH_2_–) and for the tert-butyl group (–OC(CH_3_)_3_) at δ_b_ = 1.45 ppm (the subindexes indicate the signal represented in Figure S1a).

After hydrolysis, new signals are observed for the hydroxyl group (–OH) at δ_4_ = 12 ppm (Figure S1b). The allylic group signals have no differences according to the protected copolymer, showing signs at δ_1_ = 4.5 ppm (–CH_2_–CH=CH_2_–), δ_2_ = 5.3 ppm (–CH_2_–CH=CH_2_), and δ_3_ = 5.9 ppm (–CH_2_–CH=CH_2_–). These results indicate that the signal derived from the t-butyl group at δ_b_ = 1.45 ppm disappeared due to the formation of a carboxylic group after hydrolysis. SEC measurement indicates that the copolymer has an average molecular weight of 1401 g/mol and a low polydispersity index (PDI = 1.20), as expected for a controlled polymerization process, such as ATRP synthesis.

The chemical composition of the compounds obtained in both stages was monitored via FT-IR and Raman to confirm the presence of characteristic signals related to the protection and unprotection of the copolymers. The FT-IR spectra of the copolymer obtained in stage 1 (protected) are shown in Figure S2a (Supplementary Materials, red line). This spectrum shows characteristic signals of the tert-butyl group with absorption bands located at 2980 cm^−1^ and 2930 cm^−1^, corresponding to the antisymmetric/symmetric vibrations of -CH_3_ and -CH_2_-, respectively [31]. As shown in Figure S2b (black line), these peaks disappear after hydrolysis. The same signals can be noticed in the Raman spectrum (Figure S2b). In this case, it is possible to observe a decrease in the peak intensities associated with the antisymmetric/symmetric vibrations of -CH_3_ and -CH_2_- due to the methyl group elimination. By removing the tert-butyl group, an intense and broad signal of the O-H vibration of the carboxylic group appears (>C=O or –C-COOH-stretching) between 3600 cm^−1^ and 2400 cm^−1^ (Figure S2a, black line). Similar behavior was observed for the stretching vibration of the carbonyl group (C=O) at 1730 cm^−1^. After hydrolysis, an increase in the peak width due to the formation of –COOH groups in the polymer chain could be observed [32]. The typical stretching vibration of the C=C bond of the allyl group could only be monitored through Raman at 1630 cm^−1^ [33]. In this case, no significant variation was observed between the protected and unprotected copolymer spectrum, indicating that the allyl group does not undergo any change in the reaction; this is relevant because this group serves as an anchoring point for the crosslinking agent for the upcoming hydrogel polymerization.

In conclusion, via Raman and FT-IR spectroscopies, it was possible to asseverate the copolymer formation through the ATRP technique. Together with this, the hydrolysis could be corroborated by detecting the characteristic signals of the protected and unprotected copolymers. Once the polymer synthesis was validated, the final copolymer was mixed with PEGDA_575_ as a crosslinking agent to generate a hydrogel with amphiphilic properties and enough viscosity to be deposited using the dip-coating technique.

### 3.2. Substrates Characterization

Previous studies carried out by our research group showed that these amphiphilic hydrogels (based on PEGDA) tend to form homogeneous layers on substrates when presenting a low contact angle [25]. The polar nature of the surface could be varied by various methods, such as the incorporation of self-assembled monolayers [34], plasma oxidation [35], or ozonation (UVO) [25], the latter being one of the straightforward and most effective methods. Accordingly, the different substrates were subjected to an ozonation process to promote the adhesion of the functional hydrogel.

#### Contact Angle Analysis of the Substrates

As a result of the interactions with the O_3_, the sample surface presents mainly hydrophilic groups on top. It is relevant to mention that spectroscopic analysis does not allow the detection of these small changes in the material surface. ATR-FTIR spectroscopy has a depth penetration close to 0.5 or 2 μm. Thus, few hydroxylic groups on the surface do not generate any critical change in the signals detected, making it complicated to resolve [36]. Therefore, the inclusion of hydrophilic groups could be reflected indirectly in an increased wettability of the substrates (low contact angle) [37].

The decrease of the contact angle and the irradiation time for each polymeric material after ozonation are the following: PLA = 80.56 ± 1.72° → 29.53 ± 0.65°, Δθ = 51.03°, 30 min; PCL = 68.90 ± 0.34° → 33.50 ± 0.29°, Δθ = 35.40°, 20 min; HIPS = 79.81 ± 2.12° → 22.15 ± 0.62°, Δθ = 57.66°, 30 min; and TPU = 105.7 ± 1.59° → 27.11 ± 0.42°, Δθ = 78.59°, 40 min. Interestingly, these results show that, independently of the initial contact angle of the material, all of the contact angles reach similar values close to 22–33° (hydrophilic surface) after the ozonation process.

To deeply analyze the wettability behavior of the materials, three solvents with different polar natures and surface tensions were used: water (polar), glycerol, and diiodomethane (nonpolar). Table 2 shows each sample’s dispersive and polar contribution (γ_s_^D^ and γ_s_^P^), the contact angle for each solvent, and the surface free energy (SFE) values after UVO treatment. These values were computed using the Owens–Wendt model at room temperature [38]. The surface tensions for the solvents used are: water (total: 72.8 dyne/cm; dispersive: 21.8 dyne/cm, and polar: 51.0 dyne/cm), glycerol (total: 64.0 dyne/cm; dispersive: 34.0 dyne/cm, and polar: 30.0 dyne/cm), and diiodomethane (total: 50.8 dyne/cm; dispersive: 50.8 dyne/cm, and polar: 0 dyne/cm). Thus, the total values of SFE for PLA, PCL, HIPS, and TPU are similar, ranging between ~56 and ~69 dyne/cm. As it is possible to observe from the contact angles, all of the substrates after UVO treatment present a hydrophilic surface, which is corroborated using a nonpolar liquid phase as glycerol; in this case, the contact angles were higher, ranging from ~48 to ~71°.

Interestingly, the contact angles with diiodomethane, which is also a nonpolar solvent, show low contact angles, even lower than water contact angles in some cases, indicating that the wettability of the surface is not so dependent on the polarity but rather on the surface tension of the liquid, which for diiodomethane is quite close to the water. This behavior indirectly implies that the surface morphology of the material generates much more effect on the contact angle than the polarity of the surface itself. This behavior should not surprise us since it is widespread in bio-inspired materials, such as superhydrophobicity found in lotus leaves, the gecko feet’s outstanding adhesion, or the sharkskin’s drag reduction [39,40,41]. These materials, like the ones proposed here, are based on the synergistic effects related to the interaction between the microstructure and their chemical functionality.

### 3.3. Morphological Study of the Wrinkle Patterns in the Different Substrates

Once the substrate was fully characterized, thin hydrogel layers were deposited on top using the dip-coating technique. Then, the hydrogel was exposed to UV light to polymerize the hydrogel, and then to a vacuum, and argon plasma to trigger the formation of spontaneous wrinkled patterns. These patterns were morphologically analyzed using AFM and FE-SEM methodologies.

**Figure 3:**
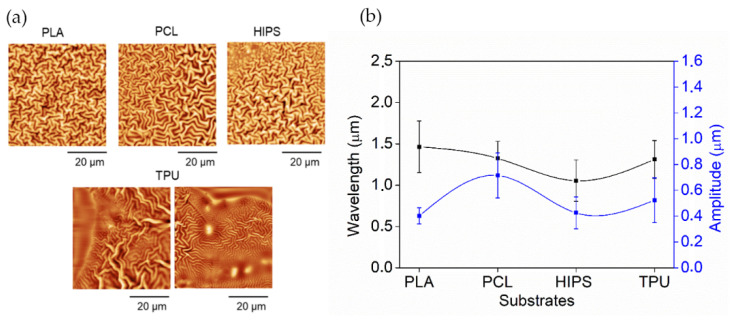
(**a**) AFM micrographs of the wrinkles pattern, using different scaffolds, and (**b**) graph of wavelength (black line) and amplitude (blue line) of the wrinkled patterns.

**Figure 4:**
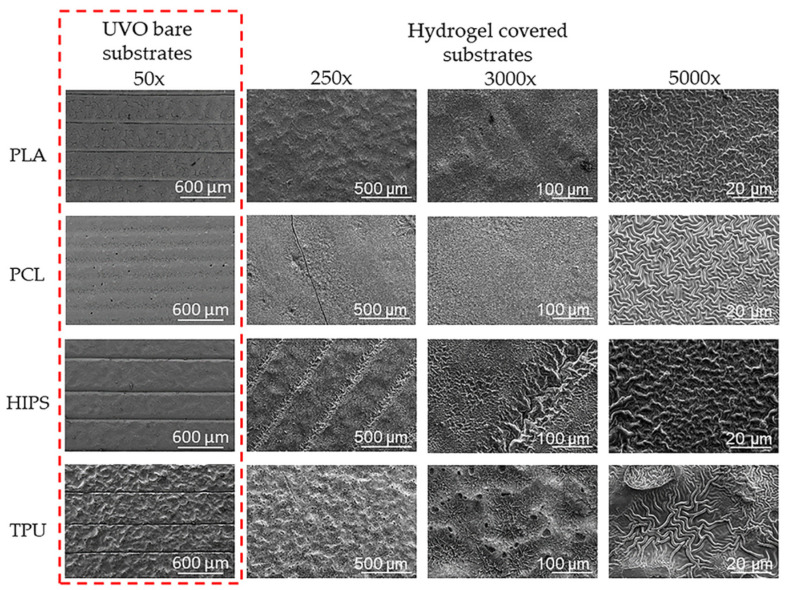
FE-SEM micrographies of the wrinkle patterns in different substrates using magnifications of 250×, 3000×, and 5000×.

Therefore, the ratio between the amplitude and wavelength (aspect ratio) of wrinkled patterns was calculated, resulting in values of 0.28 ± 0.07 for PLA; 0.54 ± 0.15 for PCL; 0.40 ± 0.15 for HIPS; and 0.40 ± 0.14 for TPU. According to these results, the aspect ratios are close and below 0.5 in all of the cases, implying that, despite the slight changes in their dimensions, they change together to maintain a similar structure during the wrinkling process. These results also indicate that the wrinkles belong to the ripples type, which corresponds to a smooth structuring pattern (with lower amplitude than the wavelength) with a homogenous distribution on the surface, as shown in Figure 3a. Only TPU shows disordered and non-homogeneous wrinkled patterns; the explication could be related to the behavior shown in Figure 4.

On the other hand, in Figure 3b, wrinkle pattern wavelength and amplitude show minor variations depending on the type of polymer used as the substrate; the wavelength varies between ~1.0 and ~1.5 μm; and amplitude varies between ~0.4 and ~0.8 μm. In the Supplementary Materials section (Figure S3), the dependency of the roughness and area increase percentage with the substrate type is depicted. In the case of roughness, much more abrupt changes could be observed, implying that despite the local changes in wrinkle shape, the variations are minimal (similar aspect ratio in all cases, according to previously mentioned results). At the same time, globally, the patterns present important changes in their shape and distribution. The latter may be because the hydrogel films are deposited on a non-flat surface, as shown in Figure 4 (first column). The irregular surface will profoundly affect the global distribution of the patterns and how they adhere to the substrate, causing changes in its thickness and, therefore, in the size and shape of the wrinkles formed on top. Increased area percentages show that despite their small dimensions, the wrinkled patterns formed on the surface of the materials allow an increase of available surface area between 12% to 20% compared to a flat surface.

Figure 4 shows the surface topography studies carried out via FE-SEM at different magnifications (250×, 3000×, and 5000×) for all substrates (PLA, PCL, HIPS, and TPU). At 250× it is possible to observe that both PLA and HIPS show a completely covered surface with some irregularities (clusters) in the surface. In contrast, PCL offers a smooth and homogenously deposited surface. TPU is the one that presents an atypical behavior. As it is possible to observe, an irregular surface with clusters could be observed on the top of the filament, while in the slits or union between the filaments, a thick layer of hydrogel and prominent wrinkles are observed. This effect could be related to the inhomogeneity of the substrate.

While PLA, HIPS, and TPU show no significant or visible change after the UVO treatment (50× magnification column in Figure 4), the PCL, which has a lower Tg than the rest of the polymers, seems more affected by the temperature generated by the UVO lamp during the treatment; thus causing a smooth surface without any irregularity. At 3000× and 5000×, the wrinkled micropatterns could be visualized; in the cases of PLA and PCL, homogenous and evenly distributed wrinkles are observed, while in the case of HIPS and TPU, the size and distribution of the wrinkles are irregular, showing pores and clusters of wrinkles with variable sizes. According to these results, PCL was selected to continue elaborating three-dimensional multi-hierarchical porous pieces due to its optimal coating and homogeneous distribution of the wrinkled patterns.

### 3.4. Bioactive Agent Synthesis

nHA was synthesized according to the methodology reported by Karampour et al. (2021) [28] and compared with a commercial product obtained from Sigma Aldrich (CAS: 12167-74-7). Via FE-SEM, ATR-FTIR, and EDX analysis, it was possible to measure the final product’s morphology, chemistry, and crystallographic structure. Morphological studies (FE-SEM) showed a spherical-like morphology for the particles, with an approximate diameter of 58.1 ± 15.1 nm for the synthesized sample and 54.1 ± 14.3 nm for the commercial nHA (Supplementary Materials, Figure S4a,b). On the other hand, the diffractogram of both samples shows the typical peaks for nHA according to the diffraction standard (RRUFF ID: R060180 or R130713). The diffraction peak positions were identified as 25.57°; 28.68°; 31.55°; 32.79°; 33.80°; 39.54°; 46.27°; and 49.26°, which corresponds to the Miller indices (002), (210), (211), (300), (202), (310), (222), and (213), respectively (Figure S4c). All indexed signals agree with those reported in the literature for a hexagonal crystal structure [42]. Finally, the sample was characterized by ATR-FTIR to confirm the presence of the characteristic functional groups of nHA. From the spectra in Figure S4d, the bands occurring at 554 cm^−1^ and 603 cm^−1^ correspond to O–P–O vibrational modes. The peaks at 1013 cm^−1^ and 1078 cm^−1^ are assigned to PO_4_^−3^ bending and stretching modes, respectively [43]. The weak absorption bands seen at 1420 cm^−1^ and 1448 cm^−1^ in the synthesized sample indicate the presence of CO_3_^−2^ [44]. These minimal signals in the spectra could be related to the annealing time of the nHA, greater annealing times should produce lower amounts of CO_3_^−2^ [43], like in this case. When comparing both spectra, there is an agreement in most of the peaks related to the vibrational modes of the functional groups of nHA.

### 3.5. Modified Filament Characterization and Printing of 3D Pieces

**Figure 5:**
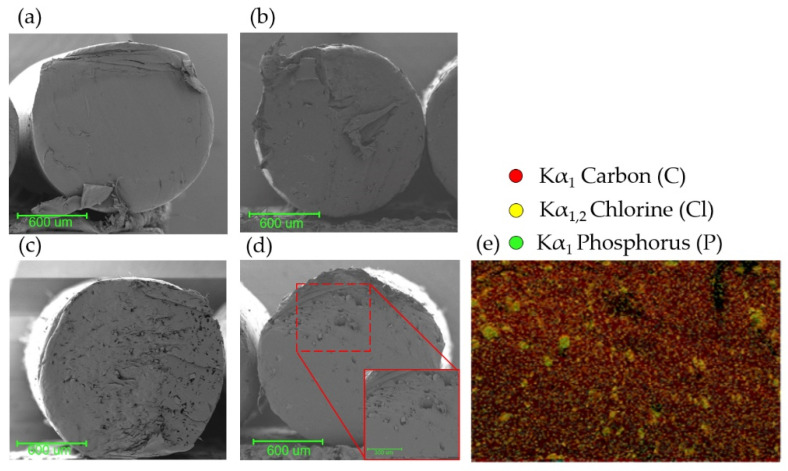
FE-SEM analysis of the filaments (**a**) native PCL; (**b**) PCL/NaCl; (**c**) PCL/nHA; (**d**) PCL/nHA/ NaCl; and (**e**) EDX elemental distribution maps of the PCL/nHA/NaCl filament.

The internal structure of the filaments was studied before printing the parts to determine the correct insertion and homogeneous distribution of the nHA and NaCl particles. The filaments were divided transversally to observe the material bulk via FE-SEM (Figure 5a–d) and EDX (Figure 5e) analysis. Figure 5a shows the natural PCL filament without modifications, which has smooth internal structures without cues or imperfections. Similarly, in Figure 5b, a smooth filament bulk can be observed. nHA particles, due to their small dimensions, do not generate a significant change in the filament’s internal morphology; thus, not producing any imperfection or visible cue. Figure 5c,d clearly shows the salt crystals of variable sizes homogenously distributed in the interior of the filament. To corroborate this assumption, an EDX analysis of the PCL/nHA/NaCl material was performed, through which it was possible to detect the atoms related to NaCl crystals (K_α1,2_ chlorine, yellow) and the signals belonging to HA (K_α1_ phosphorus, green) homogenously distributed on the entire surface (Figure 5e). Similarly, the carbon atoms (K_α1_ carbon, red), related to the organic part of the scaffold (polymeric matrix), are also homogenously distributed in the background.

**Figure 6:**
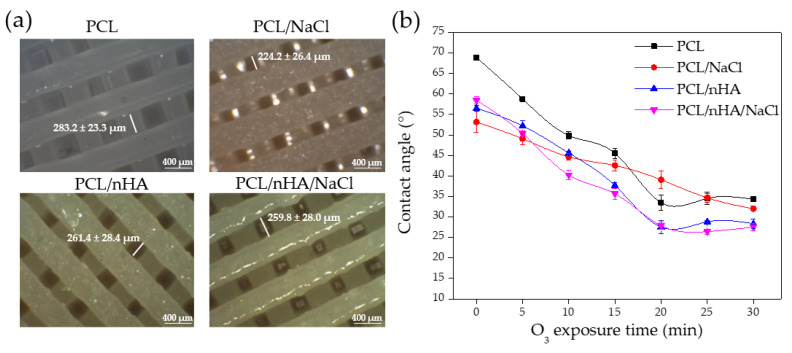
(**a**) Internal microchannel, and (**b**) graph of contact angle vs. O_3_ exposure time of the different scaffolds.

Figure S5 (Supplementary Material) shows photographs of the cylindrical pieces printed under the described conditions in the methodology. As seen in these images, it was possible to print cylindrical parts with the modified filaments. Adding NaCl and nHA particles generally produces a lower edge definition and resolution than the piece printed with the commercial PCL filament. Through optical microscopy (Figure 6a), the formation of internal micro-channels in the printed piece was analyzed. The gap between these channels changes according to the filament used in each case, from 220 to 290 µm. Mantila et al., reported the manufacturing of scaffolds based on PCL and HA with a honeycomb-like design and complete interconnectivity of the pores by FDM. The authors demonstrated that the formation of micropores with a diameter between 290 and 310 µm increases the bone regeneration rate [45]. Similar conclusions were obtained parallelly by Sohrabian et al. [16], but regarding the mechanical properties of the materials, indicating that the internal architecture of the printed part is fundamental for the overall performance of the scaffold.

Finally, in Figure 6b, a graph shows the evolution of the contact angle with the increasing UVO exposure time. All substrates show a descending tendency of contact angle with the increase in UVO exposure time, which is expected due to the interaction of the surface with the ozone.

### 3.6. Leached 3D Pieces

**Figure 7:**
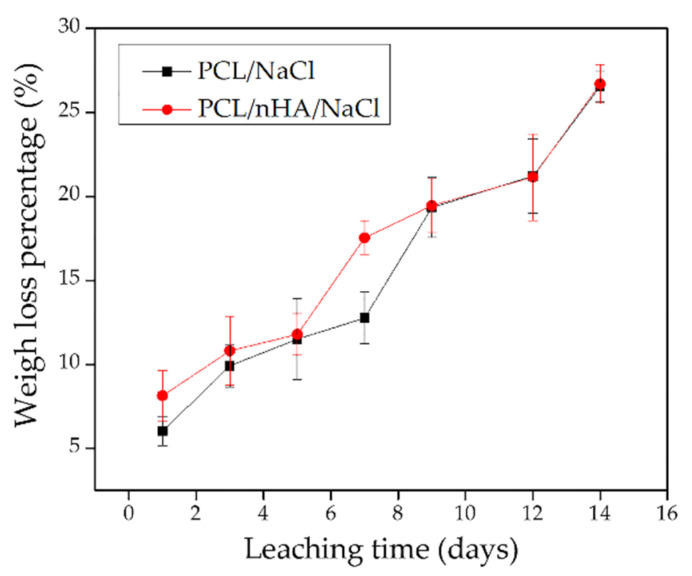
Weight loss percentage versus leaching time in PCL/NaCl (black line) and PCL/nHA/NaCl (red line) scaffolds.

Only the scaffolds with the porogen agent in their structure (PCL/NaCl and PCL/nHA/NaCl) were considered for studying the salt leaching process and their effectivity in forming pores. These pieces were leached for 1, 3, 5, 7, 9, 12, and 14 days to determine a weight loss curve. The pieces’ mass loss and surface morphology were studied on different days after being dried. As shown in the graphs (Figure 7), the weight loss tends to grow linearly concerning the leaching days. In both cases, the percentages obtained were approximately 26%, consistent with the initial concentration of salt incorporated into the filaments (30%). However, the weight loss value may be related not only to the NaCl particles but also to a loss of the bioactive agent (nHA) during the leaching process. Accordingly, and with the aim of clarifying the situation, a morphological study of the material surface was carried out.

**Figure 8:**
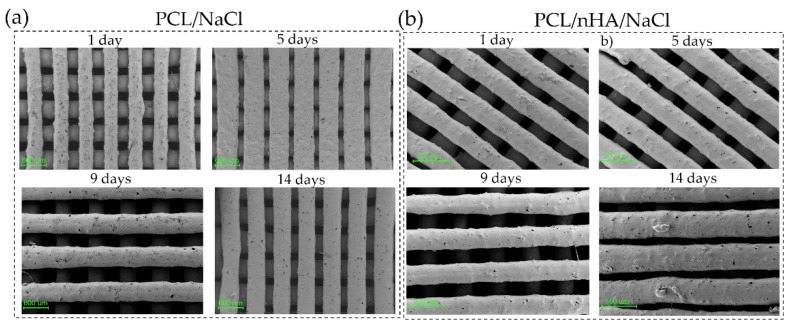
FE-SEM micrographs of pore formation at the surface level of (**a**) PCL/NaCl, and (**b**) PCL/nHA/NaCl scaffolds at 1, 5, 9, and 14 days.

The scaffold’s surfaces were micrographed with FE-SEM. The two materials were topographically analyzed: PCL/NaCl and PCL/nHA/NaCl (Figure 8). In both cases, it is possible to observe the effect that leaching time produces in the material topography. In general, the number of pores increases progressively with leaching time. Analyzing these images, it was possible to determine that the number of pores increased by ~60% from 1 to 14 days.

The PCL/nHA/NaCl samples were studied after 14 days of leaching to determine the size of the pores present on both the surface and inside the pieces. For this, a transversal incision was made to observe the internal morphology of the sample (Figure S6). Figure S6 shows pores both at the surface and at the bulk of the filament, as was already mentioned before (Figure 8). Parallel EDX measurements also demonstrate a homogenous dispersion of the nHA particles in the polymeric matrix of the PCL/nHA/NaCl leached sample (Figure S7), consistent with Figure 5 for the fabricated filament. Similar results have been reported for mixtures of PCL with HA microparticles (10 to 25 µm). The authors demonstrate that it is possible to incorporate HA particles homogeneously in the material, avoiding agglomeration or clustering. They also established enhanced material biocompatibility by including HA particles [46]. Using the images in Figure 8, the pore size could be measured manually, and the results were represented by histograms (Figure S8a,b in the Supplementary Material). By analyzing these data, it is possible to observe that the pore sizes range between 4 and 70 µm for both samples, which is consistent with the particle size (≤75 μm). Despite this wide size distribution, an average pore size of 28.14 ± 12.52 µm and 25.72 ± 12.78 µm was determined for the PCL/NaCl and PCL/nHA/NaCl samples, respectively.

**Figure 9:**
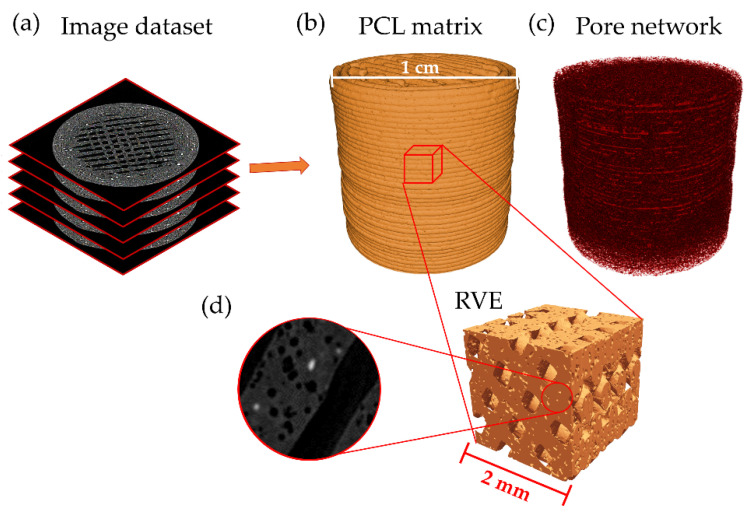
Schematic representation of microCT analysis showing (**a**) three-dimensional models; (**b**) PCL matrix; (**c**) pore network obtained from the images; and (**d**) one RVE extracted from the structure was shown with a magnified sector showing the porous filament.

To deeply analyze the effect that produces the lixiviation on the 3D printed scaffolds, microcomputing tomography (microCT) was performed on the sample PCL/nHA/NaCl. MicroCT allowed us to obtain projected images of the sample from different angles to reconstruct the internal structure of the scaffold. This dataset of transversal images created a three-dimensional model of the printed scaffold (Figure 9a). These data were used to extract geometrical information from the images, such as porosity, Euler number, or interconnectivity index. Figure 9b,c shows the three-dimensional model obtained from the PCL matrix and the pore network generated after the porogen particles’ lixiviation. Unsharp mask and Kuwahara filters were applied to these images with the aim of enhancing contrast and reducing noise from the dataset [47]. Then, a three-dimensional automatic Otsu thresholding process was used to obtain a segmented image of the material pores. As it is possible to observe from Figure 9c, the pore distribution on the material is exceptionally homogenous, showing no particular sector with a high concentration of pores. To corroborate this assumption, four cubical shaped representative volume elements (RVE) were selected from four zones (equally distanced) in the vertical direction of the printed cylinder, obtaining a total of 16 RVE for the piece (Figure 9d). These RVEs were used to determine the local porosity of each sector. By dividing the volume of the pores found in the RVE by the volume of the PCL matrix, it was possible to obtain the porosity of the filament [48]. The porosity of the filament ranged from 9.94% to 13.65% for the different RVEs, resulting in a porosity mean of 12.07 ± 1.09%. This value was close to the amount of NaCl loss after the lixiviation process according to the EDX analysis (Figure S7, Supplementary Material). Although this porosity is lower than the theoretical porosity that the filament should have (30% *w*/*w*, which corresponds to an 18.64% *v*/*v*), the filament porosity is very close to this value (Δporosity = 6.57%). On the other hand, considering the microchannels formed in the structure using the CAD model, the piece’s global porosity (lixiviated pores plus CAD-created microchannels) grows to 42.08 ± 4.78%. We suppose that this level of global porosity is enough to ensure high pore interconnectivity in the scaffold and, therefore, to encourage the cellular viability and proliferation of the material.

**Figure 10:**
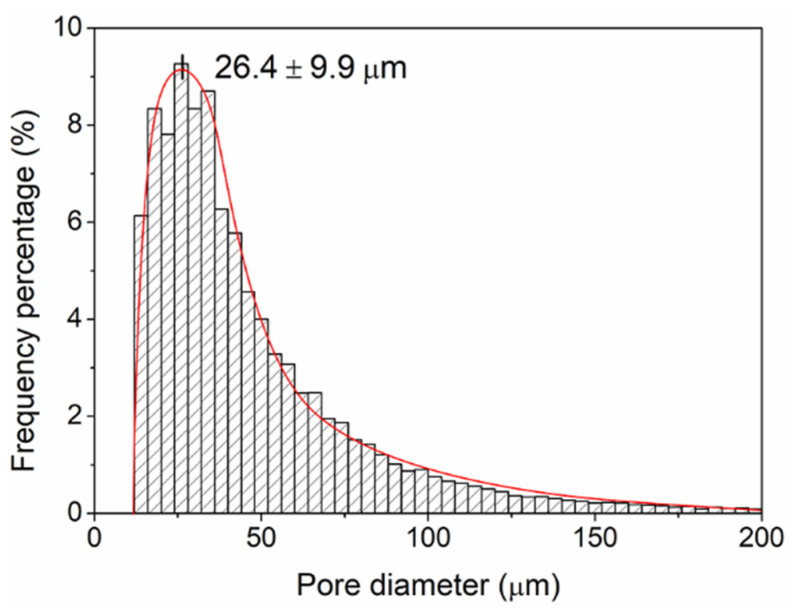
Pore size distribution of the leached scaffold determined via microCT analysis.

On the other hand, pore size distribution was also determined from these 16 RVE by computing the diameter of each pore. The surface of each pore was determined by using a 3D edge detector filter; then, by adjusting an ellipsoid on the pore surface, it was possible to determine the three principal axes of the ellipsoid, computing the pore diameter as the mean value. Figure 10 shows the pore diameter distribution obtained through this methodology with its corresponding distribution adjustment. The pore diameter shows a skewed distribution (lognormal), with this mode centered at 26.4 ± 9.9 μm. These results are in agreement with those calculated from the previous section’s SEM micrographies and with the NaCl particle size introduced in the PCL polymeric mixture.

### 3.7. Wrinkled Micropattern Analysis

**Figure 11:**
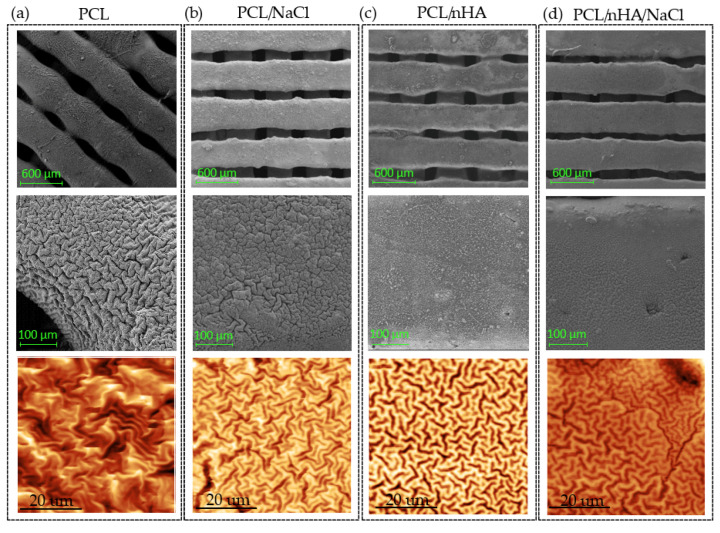
Surface analysis obtained by FE-SEM and AFM for the scaffolds composed of (**a**) PCL; (**b**) PCL/NaCl; (**c**) PCL/nHA; and (**d**) PCL/nHA/NaCl.

**Figure 12:**
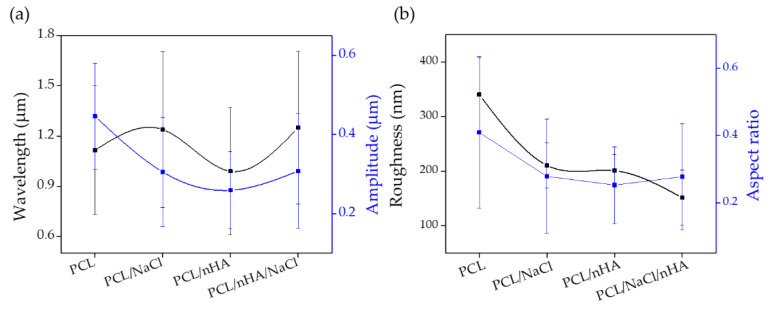
Results obtained by AFM of the *net*-poly(AAc-*co*-AMA) hydrogel deposited on the different PCL substrates: (**a**) Wavelength (black line) and amplitude (blue line) and (**b**) roughness (black line).

Once the different substrates had been treated with UVO and leached, the hydrogel was deposited using the dip-coating technique. The coated substrates were then exposed to UV (λ = 365 nm), a vacuum, argon plasma irradiation, and UV again to promote the spontaneous formation of wrinkled micropatterns according to the methodology previously proposed by our research group [17]. The morphology and topography of the hydrogel pieces were monitored by FE-SEM and AFM, respectively. As shown in Figure 11, it was possible to obtain wrinkled micropatterns with high coverture for all samples analyzed. The type of micropattern obtained is similar for the different substrates, indicating that, in this case, the material composition of the scaffold does not considerably affect the distribution and ordering of the wrinkled patterns, but the size of the wrinkles formed does differ between the different substrates. As it is possible to observe in Figure 12a, the samples have a slight variation between 0.29 µm and 0.45 µm for the amplitude and between 1.08 µm and 1.16 µm for the wavelength. In the case of the roughness (Figure 12b), a considerable variation was determined from 162 nm to 336 nm, indicating important changes in the shape and distribution of the surface are observed globally, probably due to the presence of the filaments, creating a non-planar surface in which the wrinkles varied considerably depending on the position (smooth zone of the filament or gap between filaments).

### 3.8. Cell Viability Studies

**Figure 13:**
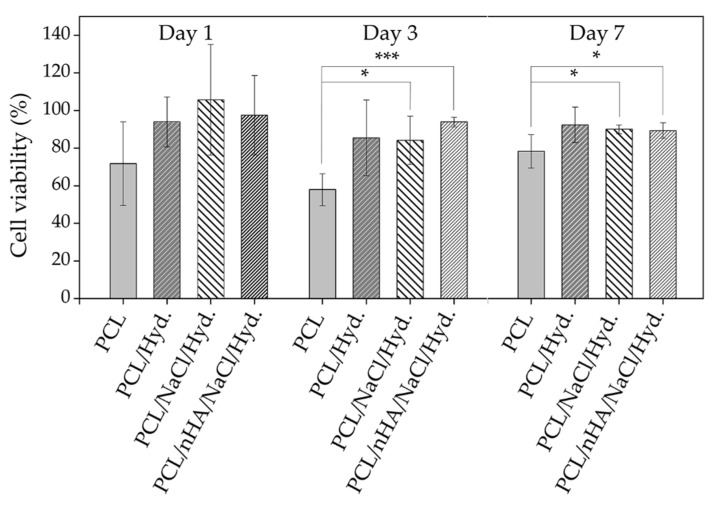
Cell viability studies obtained from AlamarBlue analysis for the samples PCL, PCL + Hydrogel, PCL/NaCl + Hydrogel, and PCL/nHA/NaCl + Hydrogel, for 1, 3, and 7 days incubation. Significant differences between the analyzed groups are indicated by * (*p*-value ≤ 0.05) and *** (*p*-value ≤ 0.001).

Once the material was utterly characterized and the formation of homogenous wrinkled patterns on the top of the scaffolds is corroborated, it becomes essential to analyze the effect on cell compatibility that generates the presence of the hydrogel on the top of the scaffold. Therefore, osteoblasts MC3T3 were cultured for 7 days, and then AlamarBlue analyses were carried out, allowing us to determine cellular viability through the metabolism activity of the cells. ANOVA statistical analysis was carried out for each group (1, 3, and 7 days) using the null hypothesis that the mean of the distributions is equal. This analysis results in *p*-values that indicate the statistical significance between the different samples, represented as asterisks in Figure 13. In all cases, the material presents an acceptable behavior regarding its biocompatibility, approaching the maximum achievable experimental value in each case (control sample). Comparing the AlamarBlue fluorescence generated by the different samples against the control (bare petri dish), it is possible to conclude that the hydrogel coating increases cellular viability when bare PCL is compared to PCL/hydrogel sample.

**Figure 14:**
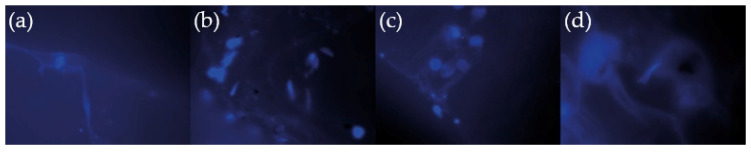
Epifluorescence micrographs of DAPI-stained MC3T3 cells seeded on (**a**) PCL; (**b**) PCL + Hydrogel; (**c**) PCL/NaCl + Hydrogel; and (**d**) PCL/nHA/NaCl + Hydrogel.

In general, the cell viability for all modified samples (PCL/hydrogel, PCL/NaCl/hydrogel, and PCL/NaCl/nHA/hydrogel) is greater than the bare polymer (PCL), indicating that these modifications make the scaffold an even more biocompatible device. In general, for 1 incubation day, no significant difference could be observed, but for 3 and 7 incubation days, the situation is different. The percentages of cellular viability comparing the PCL with the control are 78.3 ± 0.5% and 94.3 ± 0.5% for the sample of PCL/hydrogel, both at 7 days of incubation, indicating a significant difference when the hydrogel layer is included as scaffold coating (*p* < 0.05, * statistical significance). The same conclusion could be derived from the results for 3 days of incubation, showing a significant statistical difference between the bare PCL polymer and PCL/NaCl/hydrogel and PCL/NaCl/nHA/hydrogel samples. The inclusion of NaCl (porogen agent, PCL/NaCl/hydrogel) and nHA (bioactive compound, PCL/NaCl/nHA/hydrogel) in the material does not produce significant differences in the cell viability at least up to 7 days of analysis (*p* > 0.05, no significant difference Figure 13). These results are supported by studies carried out on other materials [49], where the inclusion of nHA in a polymeric mixture of poly(L-lactide) does not produce important variations in the cell viability of the scaffold. This effect could be related to two factors; firstly, the amount of nHA included in the mixture could be lower than necessary to present important variations, even more considering that a large part of this bioactive compound is contained within the bulk of the material and not on its surface. Secondly, it is possible that some of the pores formed via the leaching process could be covered by the thin wrinkled hydrogel film; thus, avoiding a proper interconnection between pores and, therefore, reducing the cell viability capacity of the material. However, epifluorescence images (Figure 14) show that the cells tend to adhere to the material surface, their filopodia and lamellipodia being detectable, which is indicative of the high material biocompatibility (almost 20% more cellular viability when the hydrogel layer is included).

### 3.9. Mechanical Tests

Finally, uniaxial mechanical tests were carried out on the samples to determine the influence of the inclusion of nHA particles and pores in the PCL matrix. PCL has a relatively poor mechanical performance, showing low tensile and compressive strengths for 3D printed parts [12], which is why nHA was included in the polymer mixture to improve its mechanical properties. Compressive tests were carried out both in quasistatic and dynamic regimes (0.05 and 0.5 mm/min), reaching compressive forces and strains of 500 N (~8–10% strain) and ~6–7 kN (~70% strain) for each case. In any case, the samples suffered a fracture due to the ductility of the PCL.

On the one hand, in the quasistatic regime analysis, no significant differences were observed between the different materials, showing similar Young modulus and maximum strain capacity in all of the cases. This could be related to two main reasons: first, due to the maximum load applied (500 N), just the elastic zone of the PCL could be reached, and second, the low strain rate for compression allows the sample to re-accommodate its internal structure to bear the load; thus, not showing any significant difference in the elastic zone (≤10% strain) for the quasistatic regime.

**Figure 15:**
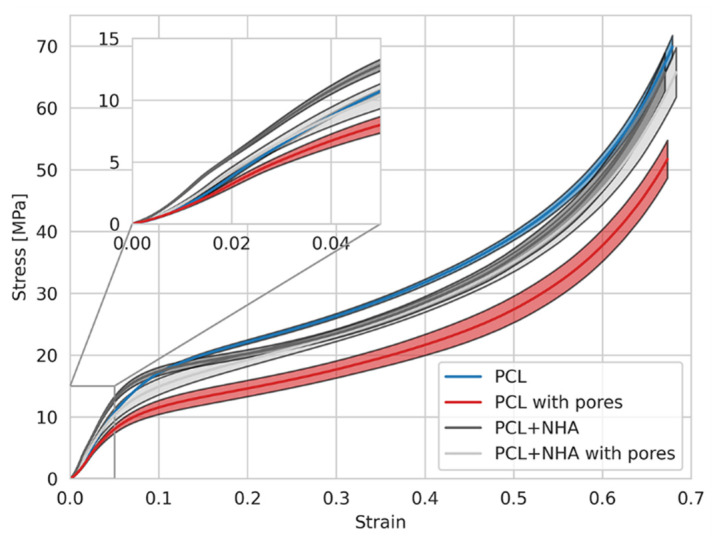
Stress–strain curves for the compressive mechanical tests carried out with the samples PCL, PCL/NaCl (PCL with pores), PCL/nHA, and PCL/nHA/NaCl (PCL + nHA with pores) in a dynamical regime. A magnification of the elastic zone could be observed.

On the other hand, in the dynamic regime, the maximum load is considerably higher (~6–7 kN), reaching a maximum of ~70% strain, enough to reach the viscoelastic zone. Figure 15 shows the results obtained in this case. As it is possible to observe, the elastic zone of the stress–strain curve (subplot in Figure 15) demonstrates that the inclusion of nHA in the mixture (gray curve) increases the mechanical performance of the bare material (blue curve). In contrast, the inclusion of pores (lixiviated NaCl) produces materials with poor mechanical performance (light grey and red curves), namely PCL with pores (PCL/NaCl), the material with the lower Young modulus (Table 3). These results are expected because including pores in the structure means removing material; thus, decreasing its mechanical performance against compressive stress. The material’s behavior in the viscoelastic zone (strain higher than ~10%) is interesting. All of the samples show a yield point and a strain softening behavior followed by a strain hardening, typical for thermoplastics, but, while the samples PCL and PCL/NaCl (PCL with pores) present a similar curve with different stiffness, the samples PCL/nHA and PCL/nHA/NaCl (PCL + nHA with pores) slightly decrease their mechanical performance; thus, PCL is the stiffest sample at higher strains. These results indicate that at low strains (≤10% strain, elastic zone), the nHA inclusion generates increasing mechanical performance, but for high strains (between 10% and 70%), the nHA inclusion in the mixture results in the deterioration of the mechanical resistance of the material. This effect, commonly known as the Payne/Mullins effect [50], is characteristic of polymer blends filled with nanoparticles. It can be attributed to the deformation-induced changes in the material’s microstructure, i.e., breakage and recovery of weak physical bonds linked to adjacent particle clusters.

Focusing on the elastic region of the stress–strain curve, a direct relationship between applied loads and resulting strains is experienced in a deformable solid. The impact of the enhanced Young’s modulus of the PCL/nHA samples regarding the rest of the material under the same mechanical load would produce approximately 10% less strain in a pure PCL material and 40% less strain than a porous PCL structure. For example, at a load of 10 MPa, a pure PCL sample would experience a deformation of 3.88%, while the sample PCL/nHA under the same load would result in a strain of 3.52%, and finally, a porous PCL solid would result in a strain of 4.92%. Different authors also obtained similar results using PCL and bioactive particles [51,52].

Finally, although the porous PCL/nHA structure presents almost the same low-strain elastic modulus as a pure PCL scaffold, it could serve the purpose of maintaining the base elastic properties of the main material while, at the same time, its microstructure allows for increasing cell viability, as seen in the previous cell viability section.

## 4. Conclusions

This study aims to fabricate biocompatible scaffolds for bone replacement using PCL as a polymeric base and different methodologies (CAD-modeling, FDM, salt leaching, bioactive particle inclusion, and surface micropatterning) to impart a hierarchical microstructure that resembles natural bone structure.

First, the copolymeric mixture was deposited using the dip-coating technique over four types of ozone-treated polymeric substrates (PLA, PCL, HIPS, and TPU). These substrates were subjected to external stimuli to create the wrinkles, resulting in variable morphologies and covertures depending on the substrate, the most homogeneous and reproducible being PCL. Accordingly, PCL was selected to create a filament including NaCl and nHA particles in their mixture as porogen and bioactive compounds, respectively. The filament was characterized using FE-SEM and EDX to corroborate the correct insertion of both agents homogenously throughout the material. Then, cylinders were 3D printed using FDM and leached under constant stirring in deionized water to eliminate the porogen particles leaving homogenously distributed pores in the printed piece. Finally, FE-SEM and micro-CT were carried out to corroborate the size and distribution of the pores, and mechanical tests were conducted to analyze the compressive capacity of the printed cylinders. Cell cultures using MC3T3 osteoblasts were also carried out to measure cell viability over the scaffolds at 1, 3, and 7 days of incubation.

The results demonstrate that homogenously distributed pores with a mean pore size of 26.4 μm could be found in the internal structure of the printed pieces, resulting in a filament porosity of ~12% (pores in PCL filament) and a global scaffold porosity of ~42% (pores and microchannels in the whole piece). Furthermore, by using PCL as substrate, it was possible to obtain high hydrogel coverture and homogeneity, forming regular and well-distributed wrinkled micropatterns on the surface. Interestingly, the inclusion of nHA particles during filament fabrication considerably affects the material’s mechanical performance, increasing its Young modulus by more than 10%. However, it does not improve the cell viability of the osteoblasts, even though it is considered a bioactive compound that should affect the biocompatibility and the cell growth rate on its surface. This could be related to the low amount of nHA exposed in the surface material; thus, maintaining the major amount of particles in the bulk of the filament due to the deposition method (hot extrusion). Nevertheless, the cell viability of the cylinders that include the microstructured hydrogel thin film on their surface seems considerably affected by its presence. Moreover, the cell viability reaches similar values when the hydrogel-covered scaffolds are compared with the control (bare petri dish), and increasing values when compared with the bare PCL used as the substrate, indicating that the synergy between the nHA and hydrogel considerably increases cell growth and its survivor even for 7 days of incubation.

## Figures and Tables

**Table 1:** Variation of filament manufacturing parameters.

Filament	PCL (g)	nHA (g) 10%	NaCl (g) 30%
PCL	40.0	0	0
PCL/nHA	40.0	4.5	0
PCL/NaCl	40.0	0	16.0
PCL/nHA/NaCl	40.0	4.5	18.0

**Table 2:** Contact angle of the substrates and total surface free energy (SFE) values.

Substrate	Contact Angle (°)			Surface Tension Components (dyne/cm)		Total, SFE (dyne/cm)
	Water	Glycerol Anhydrous	Diiodomethane	γ_s_^D^	γ_s_^P^	
PLA	29.53 ± 0.80	50.91 ± 0.64	23.53 ± 0.80	38.70	26.95	65.65
PCL	33.50 ± 1.00	55.81 ± 1.77	23.11 ± 0.72	37.32	21.54	58.86
HIPS	22.15 ± 2.41	47.89 ± 2.39	11.70 ± 0.80	40.98	28.40	69.38
TPU	27.11 ± 0.68	71.15 ± 0.23	22.80 ± 0.43	22.65	33.81	56.46

**Table 3:** Young modulus of the different materials analyzed.

	Young Modulus (Mpa)
PCL	257.15 ± 7.70
PCL/nHA	283.68 ± 16.84
PCL/NaCl	202.94 ± 22.40
PCL/nHA/NaCl	253.84 ± 25.27

## Supplementary Materials

The following are available online at https://www.mdpi.com/article/10.3390/polym14194041/s1, Table S1: Parameters for the deposition of the copolymeric mixture on 3D substrate; Figure S1: ^1^H-NMR and structure of the (a) protected and (b) unprotected copolymer in a mole ratio of 90:10; Figure S2: (a) FT-IR, and (b) Raman spectroscopy of unprotected and protected copolymers in a mole ratio of 90:10; Figure S3: Graph roughness (line black) and area increase (line blue); Figure S4: Analysis and characterization of synthesized and commercial nHA particles. (a) FE-SEM commercial nHA. (b) FE-SEM nHA synthesized. (c) ATR-FTIR commercial nHA (line black) and synthesized (line red). (d) Analysis XRD nHA commercial (line green) and synthesized (line blue); Figure S5: Different scaffolds printed in FDM; Figure S6: FE-SEM of the internal structure of the PCL/nHA/NaCl scaffolds with 14 days of leaching in different zones; Figure S7: EDX measurements of the PCL/nHA/NaCl leached sample; Figure S8: Histogram of pore size of the: (a) PCL/NaCl and (b) PCL/nHA/NaCl scaffolds.
