# Supplementary material for: Fabrication and Testing of Multi-Hierarchical Porous Scaffolds Designed for Bone Regeneration via Additive Manufacturing Processes
Source: Polymers (Basel). 2022 Sep 27;14(19):4041. doi: 10.3390/polym14194041 (PMC9571634; doi:10.3390/polym14194041)
Supplement: Supplementary file 1 [file polymers-14-04041-s001.zip › polymers-1903724-supplementary.pdf]

Supplementary Material

Table S1. Parameters for the deposition of the copolymeric mixture on 3D substrate.

Material	Mixture deposition via dip-coating			Wrinkles patterns formation	
	Speed (mm/s)		Immersion cycles	Irradiation UV light	Vacuum time
	Input	Output			
PLA	1.10	0.45	2	30 s	1 h
PCL	1.10	0.45	2	1 min	2.5 h
**PCL/NaCl**	**1.10**	**0.45**	**2**	**1:30 min**	**2.5 h**
**PCL/nHA**	**1.10**	**0.45**	**2**	**1:30 min**	**3 h**
**PCL/nHA/NaCl**	**1.10**	**1.20**	**3**	**1:30 min**	**3 h**
HIPS	1.10	0.45	2	5 min	8 h
TPU	1.10	1.10	3	1 min	2 h

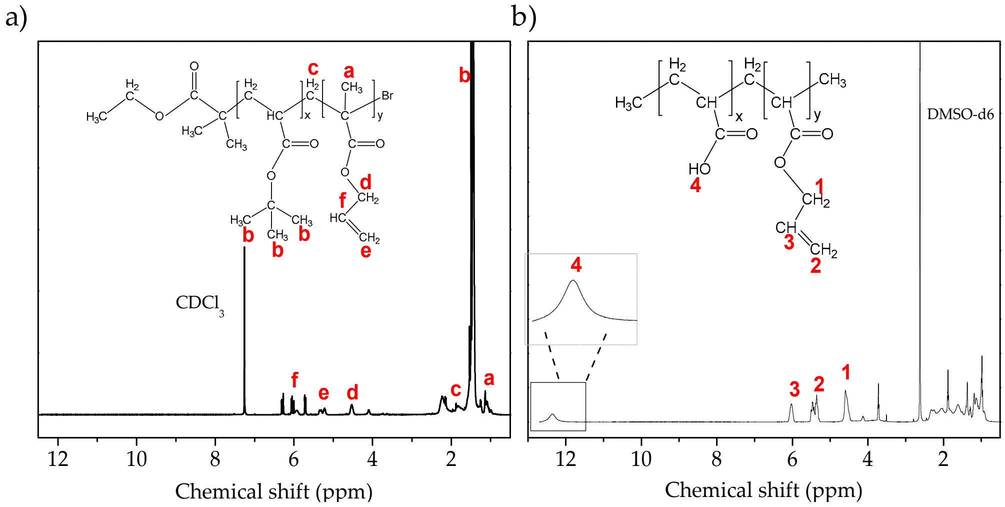

Figure S1. ^{1}H-NMR and structure of the a) protected and b) unprotected copolymer in a mole ratio of 90:10.

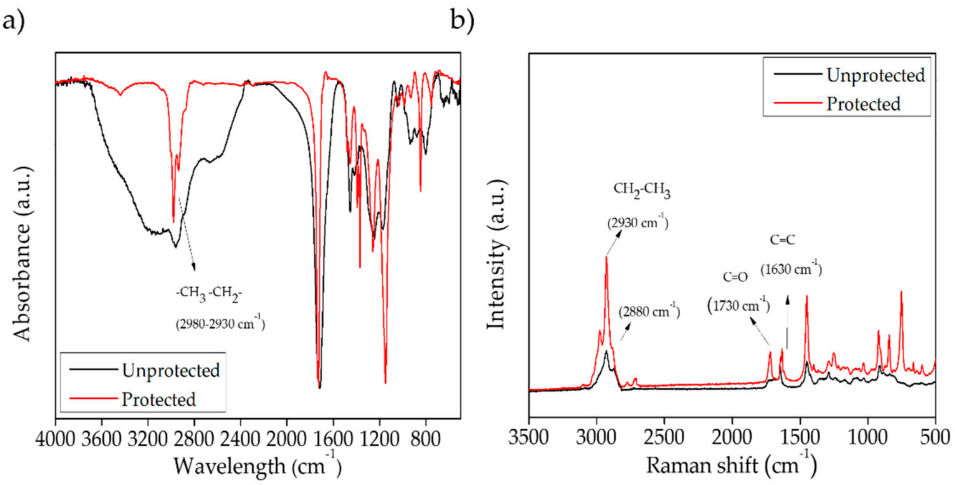

Figure S2. a) FT-IR, and b) Raman spectroscopy of unprotected and protected copolymers in a mole ratio of 90:10

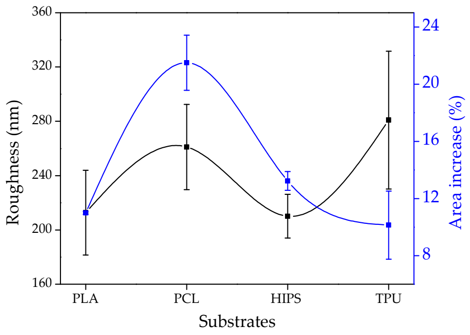

Figure S3. Graph roughness (line black) and area increase (line blue).

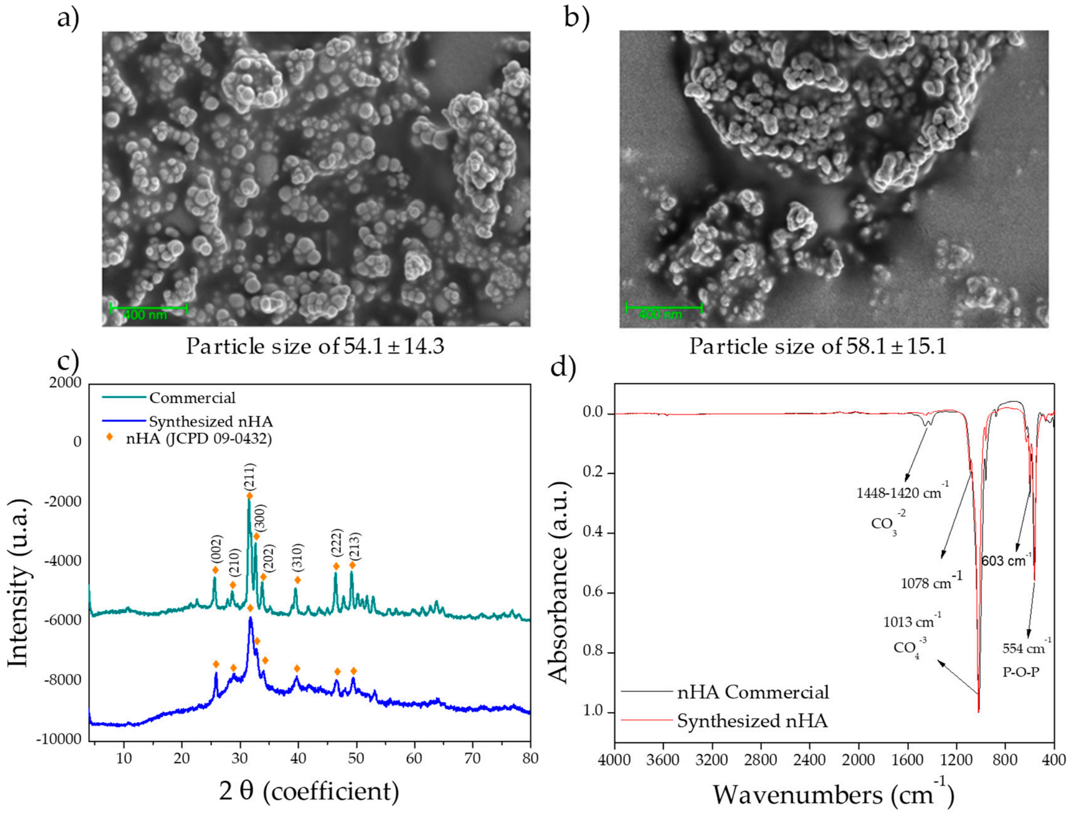

Figure S4. Analysis and characterization of synthesized and commercial nHA particles. a) FE-SEM commercial nHA. b) FE-SEM nHA synthesized. c) ATR-FTIR commercial nHA (line black) and synthesized (line red). d) Analysis XRD nHA commercial (line green) and synthesized (line blue).

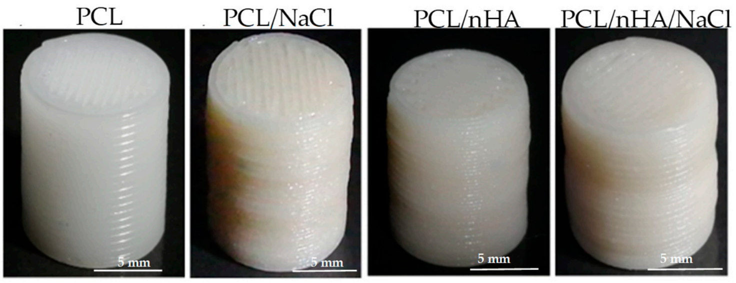

Figure S5. Different scaffolds printed in FDM.

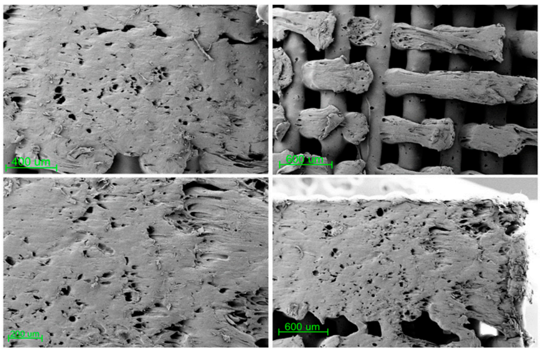

Figure S6. FE-SEM of the internal structure of the PCL/nHA/NaCl scaffolds with 14 days of leaching in different zones.

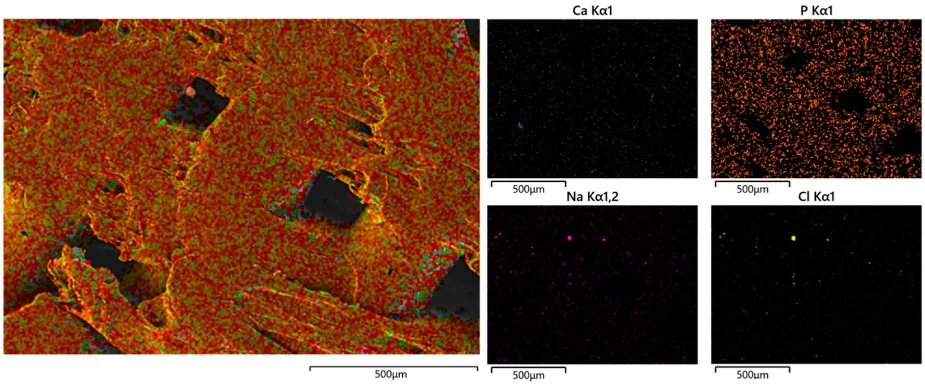

Figure S7. EDX measurements of the PCL/nHA/NaCl leached sample

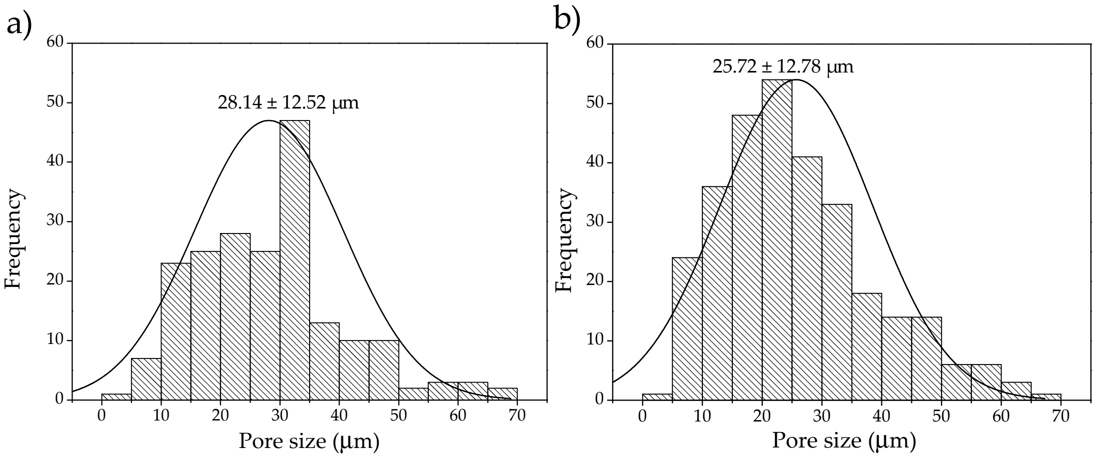

Figure S8. Histogram of pore size of the: a) PCL/NaCl and b) PCL/nHA/NaCl scaffolds.
